# The Innovative and Evolving Landscape of Topical Exosome and Peptide Therapies: A Systematic Review of the Available Literature

**DOI:** 10.1093/asjof/ojae017

**Published:** 2024-03-19

**Authors:** Makenna Ash, Meira Zibitt, Orr Shauly, Ambika Menon, Albert Losken, Daniel Gould

## Abstract

Topical antiaging therapies provide noninvasive delivery of active therapeutics. Exosomes, or extracellular nanovesicles, and peptides, small strings of amino acids, have shown promise as topical therapies in early trials, but neither is FDA approved. This review aims to elucidate the current and future landscape of topical exosomes and peptides as therapeutics for skin rejuvenation. A literature search was conducted using the keywords “peptides” OR “exosomes” AND “skin” OR “rejuvenation.” Primary endpoints included mechanisms of action in humans or live animals as well as clinical data supporting the use of exosomes or peptides topically for skin rejuvenation or wound healing. Secondary endpoints were safety, side effects, and efficacy. The articles were collected, organized, and sorted using the Covidence software (Melbourne, Australia) for systematic review. Nine articles evaluating topical application of exosomes and 9 of peptides met inclusion criteria. Topical exosomes were found to increase collagen deposition, accelerate wound healing, and improve overall cosmesis. Several clinical trials are currently underway. Topical peptides were found to improve appearance of fine lines and wrinkles, elasticity and viscoelasticity, skin texture, skin thickness, and the potential for accelerated wound healing. Peptides are quite common in “cosmeceutical” products, and several patents have been filed for topical peptide products aimed at increasing skin rejuvenation. This could indicate a movement toward pursuing FDA approval. The future of topical exosome and peptide products for the purpose of skin rejuvenation appears promising. Preliminary data from the studies reviewed here indicates that these products have the potential to be safe and effective.

**Level of Evidence: 3:**

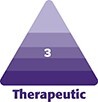

The United States population is aging. In 2020, 1 in 6 US Americans was over 65, representing a 38.1% increase over the previous 10 years.^1^ As the population continues to age, there is an increased effort to identify products that can be used to prevent signs of aging, such as wrinkles or solar spots, and rejuvenate skin through increased elasticity and hydration. Products that can be applied topically are preferred due to easier consumer accessibility and use. Topical products are less invasive than those that are injected or consumed.^2^ However, use of topical products requires unique considerations for methods of drug delivery due to difficulty penetrating the skin barrier. Therefore, active ingredients in topical medications can be less effective than in other methods of drug delivery.

As the interest in topical antiaging therapies has grown, exosomes and peptides have shown some promise in early trials. However, there are no FDA-approved topical therapies as of yet, using either peptides or exosomes as the primary ingredient. In this article, we investigate the current landscape around topical exosomes and peptides for skin rejuvenation, the available data on effectiveness, and the outlook for the future of these therapies.

Exosomes, extracellular nanovesicles, emerged into scientific literature in the 1960s by Bonucci and Anderson, described as extracellular vesicles produced by cell membrane budding. They may contain anything from lipids to proteins or nucleic acids, with the function of cellular signaling as well as waste management.^3,4^ Since these early descriptions, exosomes have been investigated for their roles in everything from cancer biology to immunology, atherosclerosis, and neurology. Their molecular roles are vast due to the potential for communication between adjacent cells, modulation of protein expression, control of cellular life cycles, influence on cell behaviors, establishment of cellular polarity, and remodeling of the extracellular matrix.^5^

Exosomes became of interest in the skincare industry due to their ability to alter the extracellular matrix, induce cell regeneration, influence the cell cycle, and target drug delivery. They have been found to have numerous cosmetic benefits, including wound healing, hydration, texture improvement, antiaging effects, and improvements in discoloration^6^ (Figure 1). Exosomes can be generated from any cell line, but it has been established that stem cell exosomes are better able to induce cell proliferation, regeneration, and wound healing and, therefore, are better fit for improvements in skin aging.^7^ Many of these benefits are seen in the injection of exosomes derived from stem cells. Less research has evaluated the cosmetic outcomes of topical application of exosomes, which is the focus of this review. Exosomes are currently not FDA approved for cosmetic use and are, therefore, unregulated as of now. Research into the use of exosomes is at the forefront of cosmetic medicine, in addition to the numerous uses of exosomes in general medicine.

**Figure 1.**
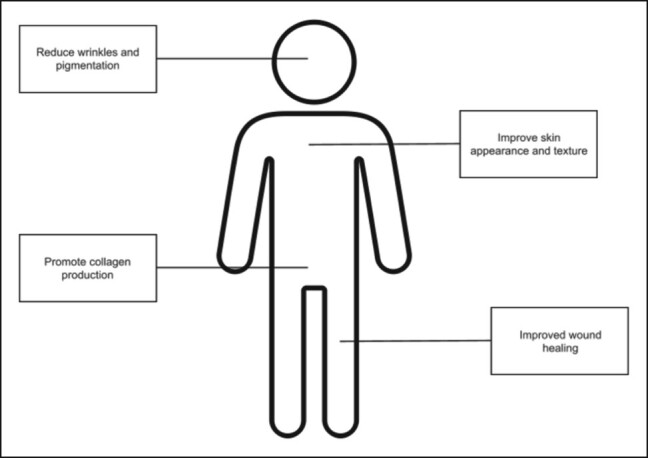
Diagram of the potential uses and benefits of topical exosome therapy in patients.

Topical peptides have gained popularity in the skincare industry for their potential benefits in promoting skin health and rejuvenation. Peptides are strings of amino acids that are used in the body to build proteins, such as collagen, which are essential for skin structure and elasticity. Peptides, as injectables, have demonstrated health benefits in inflammation, wound healing, and antimicrobial defense, and substantial research is being done evaluating the therapeutic potential of peptides for skin rejuvenation.^8^ Peptides may have the ability to stimulate collagen production, improve skin texture, and reduce the appearance of fine lines and wrinkles. Topical peptides have been used in commercial dermatology and cosmetic formulations for some years; however, there is not currently a medical-grade FDA-approved topical peptide on the dermatology market.

A hurdle to the use of topical peptides is their questionable efficacy in penetrating the skin barrier. For active ingredients to be able to affect skin texture and appearance, they must be absorbed into the dermis in a stable form. As of the time of this review, there are hundreds, if not thousands, of cosmeceuticals on the market containing peptides for the stated purpose of rejuvenating skin. Although there is substantial evidence investigating oral consumption or injection of peptides for skin rejuvenation and wound healing, there is a paucity of data, especially studies published since 2010, supporting the efficacy of peptides applied topically for the purpose of rejuvenating skin.

This review aims to elucidate the current landscape and future of topical exosomes and peptides as therapeutics for skin rejuvenation, including future FDA approval. A formal systematic review was done following the PRISMA guidelines, and discussion was supplemented using additional sources identified as being pertinent that did not fall into the inclusion/exclusion criteria of the formal review.

## METHODS

**Figure 2.**
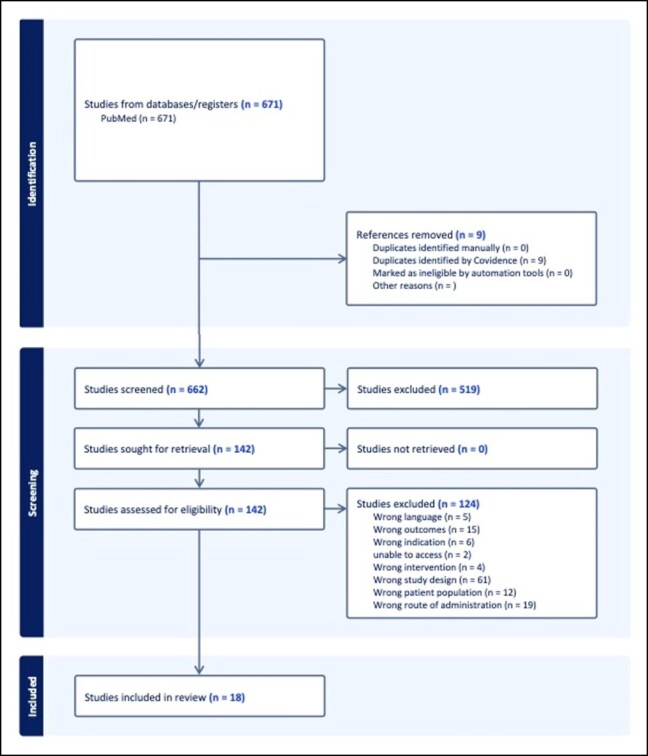
Systematic review of the literature flowchart and selected studies.

An initial search of the literature was conducted on PubMed using the keywords “peptides” OR “exosomes” AND “skin” OR “rejuvenation.” The initial search found 662 distinct articles. The parameters for the search were articles that had been published since 2010 and English. The search was run on August 15, 2023. The primary endpoints of the review were investigating mechanisms of action in humans or live animals, as well as clinical data supporting the use of exosomes or peptides topically for skin rejuvenation or wound healing. Secondary endpoints of the review were safety, side effects, and efficacy. The articles were collected, organized, and sorted using the Covidence software (Melbourne, Australia) for systematic review (Figure 2).

Inclusion criteria were: studies conducted on humans or nonhuman animals, topical application of therapy, articles published between 2010 and 2023, originally published in English, skin rejuvenation or wound healing as a primary outcome, and measureable or quantitative outcomes. Studies were excluded if they met any of the following criteria: nontopical method of delivery, in vitro study, case studies/case reports/editorials/commentaries/literature reviews/systematic reviews, full text not available, or duplicates. If a literature review or systematic review was found to be relevant, then the cited articles were included in the original data pull.

This systematic review is evaluating the clinical data supporting products that have not yet been approved by the FDA. Therefore, the results of this review alone should not be used to inform clinical judgment pending FDA approval of the products discussed herein. It is the authors' goal to evaluate the available data and the current clinical landscape in order to provide insight into the future of these therapies, and to assist the reader in their own clinical decision-making process.

## RESULTS

### Exosome Outcomes

The primary outcomes of the articles included in this literature review were wound healing and re-epithelialization, collagen deposition, inflammatory markers, facial aging (including wrinkles, radiance, and firmness), and photoaging. Exosomes were derived from various cell lines, including human fetal fibroblasts, acellular Wharton's Jelly, adipose stem cells, mesenchymal stem cells, and human platelet extract. Exosomes were all administered topically, as per the inclusion criteria, but a single study topically administered exosomes with sponge spicules^9^ and 1 study applied a topical gel matrix seeded with exosomes.^10^ Of note, 1 study also assessed exosome treatment with or without the coadministration of botulinum toxin A.^11^ One study also enriched exosomes with siRNA used to knockdown molecules, such as NF-kB.^12^

Exosomes were generally isolated by harvesting the supernatant from the cell culture growth medium, centrifuging the medium at sequentially increasing forces, filtering, and resuspending the resultant pellet. Nanoparticle tracking analysis, transmission electron microscopy, and western blots are commonly used to determine the character, morphology, and protein content of the exosomes.

### Exosome Product Availability

The studies included in this systematic literature review did not include any commercially available products. Some studies did utilize topical exosome application in conjunction with commercially available skincare products, most commonly moisturizers or SPF.

### Exosome Safety and Efficacy

Five studies determined that exosome-treated wounds showed accelerated wound healing and re-epithelialization rates or a smaller wound at some interval after treatment compared with controls.^10-14^ Three studies found that exosome-treated wounds had increased collagen deposition, with 1 study finding that, in addition to increased collagen deposition, the exosome-treated group had improved collagen alignment.^10,11,14^ Three studies found increases in inflammation in exosome-treated skin, although 1 study noted this was transient and resolved within 72 h of topical application with sponge spicules.^9,11,13^ One study found decreased inflammatory cytokines, although the exosomes contained siRNAs targeting inflammatory regulators.^12^ One study found increased polymorphonuclear leukocytes in the exosome-treated group,^13^ and Lu et al found that the exosome-treated group had decreased macrophages at the wound site.^12^ Two studies found that treatment with exosomes caused increased angiogenesis.^10,11^ Lastly, 2 studies found improvement in granulation in exosome-treated groups.^10,13^

In the discussion of cosmesis improvement, Ye et al found that exosome treatment improved sensitive skin scoring of roughness, scaling, erythema, as well as burning, tension, itching, and dryness, as assessed by 1 professional dermatologist on an objective and subjective sensitive skin index.^16^ Proffer et al found that exosome treatment produced improvements in facial aging, such as erythema, color evenness, luminosity, wrinkling, and firmness, as measured by computer analysis of photo documentation.^15^ Both of these studies excluded anyone who had prior dermatologic or cosmetic procedures, excluding a large population that would potentially be interested in this type of product. Proffer et al also utilized a twice-daily skin regimen in addition to the use of exosomes, including products such as a repair serum, SPF, and specific moisturizers which may further improve skin outcomes.^15^

### Federal Drug Administration: Exosomes

No studies of exosome therapies included in this systematic literature review are currently FDA approved or undergoing an FDA-approved clinical trial. Please see the Discussion section for further elaboration.

### Peptides Outcomes

**Figure 3.**
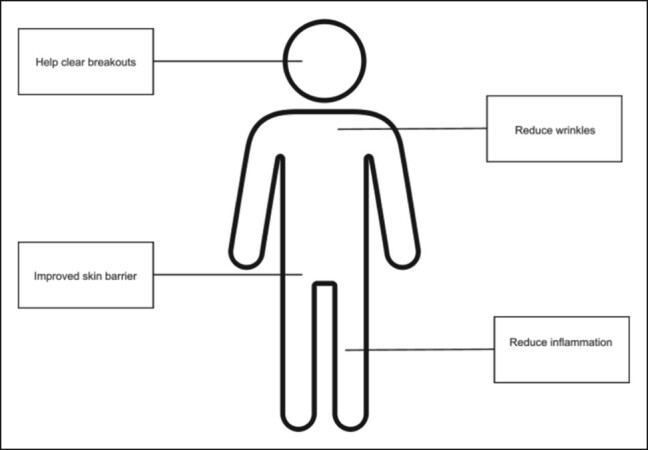
Diagram of the proposed benefits of topical peptide therapy in patients.

The primary outcomes of the studies in the reviewed outcomes were wound healing, skin rejuvenation, and resolution of photoaging. Of the peptides investigated in the reviewed articles, 4 were derived from rice proteins, 3 were derived from marine collagen extracted from sea life such as fish or oysters, and 3 referred only to the product as “peptides” with no source specification. Methods for deriving the peptides included using acid to solubilize and extract the collagen proteins from tilapia skin, fermenting rice grains with *Lactobacillus* and extracting peptides from the remaining protein, and hydrolyzing oyster protein. The peptides were then prepared in a lipophilic suspension or a hydrogel to facilitate penetration of hydrophilic peptides into the lipophilic external barrier of the skin (Figure 3). In 1 study, the peptides were combined into a sunscreen formulation also containing ascorbyl tetraisopalmitate.^17-26^

### Peptide Products

Of the assessed studies, 1 investigated a commercially available peptide product. This product was an ampoule containing peptides sourced from rice and lupin, Vitamin C, hyaluronic acid, and volcanic mineralizing water.^17^ Formulations of other peptide products investigated were varied. Some trials added peptides to an existing moisturizing solution. Others combined these peptides into a water-based solution, or even hydrogels.^18-26^

### Peptide Safety and Efficacy

Three of the assessed studies found topical peptide interventions to improve the appearance of fine lines and wrinkles. Two found these interventions to improve elasticity and viscoelasticity, although 1 found no difference in elasticity and viscoelasticity. Three found improvement in skin texture, and 2 found potential for increased wound healing. One study found an increase in skin thickness, and 3 found improved skin appearance and decreased markers of photoaging. None of the assessed studies observed any negative side effects in human participants.

### Federal Drug Administration: Peptides

There are no FDA-approved topical peptide products currently available. Since 2017, several patents have been filed in the United States and internationally for various cosmetic peptides for improving skin rejuvenation and use of the same.^27-30^ This could mark a growing interest in the field of topical bioactive peptide application, specifically for skin rejuvenation.

## DISCUSSION

### Topical Exosomes: Proposed Mechanisms of Action

As previously noted, exosomes have many potential mechanisms of actions, varying based on the cell that secretes them as well as their target and the content of the exosome itself, which can be highly variable. The various biologic materials present in the exosome, ranging from nucleic acids, to proteins or lipids and more, play a large role in determining these interactions and cellular communication. This is further complicated by the various extravesicular proteins that may be present on the exosomal surface.

The biggest challenge for topical treatments to be effective is whether or not they are able to permeate the lipophilic skin barrier. As exosomes are derived from cell membrane budding, the hypothesis is that these extracellular vesicles are able to merge with the cellular membrane of dermatocytes, and in doing so, deliver their active product into the interior of the skin cell.

Topical exosomes may affect skin rejuvenation through modulation of transforming growth factor beta (TGF-B), mitogen-activated protein kinase, and extracellular signal-regulated kinase, which all play roles in cell differentiation, cellular proliferation, and apoptosis regulation.^31,32^ Modulation of cytokines, such as TGF-B, leads to differences in extracellular matrix makeup, production of collagen, and collagenases, as well as mediates the inflammatory response to tissue damage. Increasing collagen production and modulating collagenase production can result in improved wound healing and scar formation as well as skin plumpness and elasticity. Exosomes also may mediate the inflammatory response to skin damage, improving wound healing, while promoting angiogenesis, tissue remodeling, and extracellular matrix deposition.^33^ Additionally, these cytokines play an integral role in many dermatologic processes, leading to potential for exosome use in treatment of dermatologic pathologies such as dermatitis, psoriasis, and wound healing, among others.

### Topical Exosomes: Quality and Safety Considerations

There are currently a wide range of exosome therapies under investigation. According to clinicaltrials.gov, there are hundreds of ongoing exosome-related clinical trials across a wide range of medical specialties, indicating a recent increase in interest around exosomes. The primary outcomes of current trials range from the use of exosomes as biomarkers and drug delivery systems to therapeutics and vaccines.^34^ Currently, there are FDA-approved clinical trials investigating exosome-based products for conditions such as atopic dermatitis, skin rejuvenation, psoriasis, alopecia, epidermolysis bullosa, and diabetic wound healing.

One consideration, when looking to the future of exosome-based medical treatments, is the significant overlap with stem-cell-based treatments, as many exosome therapies are derived from stem cell lines. The FDA released a warning against the use of stem-cell-derived therapies as well as a specific Public Safety Notice addressing exosome products in 2019.^35^ This warning addressed an increase in false claims that exosomes are not held to the same rigorous standards as other drugs and biologics.^36^

The exosomes examined in this systematic review were harvested through many different methods, and exosomes under investigation for other therapeutic reasons may be harvested in different manners still.^6-16^ Isolation and purification is typically done by ultracentrifugation by either differential or density gradient. Immunoaffinity is occasionally used by utilizing exosome markers, but these markers are not present on all exosomes and, therefore, are not a perfect solution. Concern for altering exosomes due to chemical interactions with the reagents must also be considered. Because of these production and quality control issues, mass production is a significant consideration when aiming to produce commercially available cosmetics. When considering dosing, many factors unique to biologics must be considered, including cellular uptake, off-target interactions, biodistribution, and exosome half-life, in addition to other pharmacodynamics and pharmacokinetic properties to consider.^36^ Many current studies are working to address these issues.

The future of exosome-based medical treatments is promising, although the FDA approval process is lengthy and some aspects of exosome-based therapies raise unique challenges. Important considerations include purification and reliability issues, production optimization and tissue-specific delivery, standardization of dosing and potency, and long-term safety questions. The heavy reliance on cell culture and the ability to reliably extract and purify the exosome product is a relatively low-yield process. Additionally, different cell lines have different culture requirements and have cell-line-specific senescence rates. Because of this, techniques to extract exosomes from 1 cell type are not likely to be the solution for others.^37^

### Topical Exosomes: Implications in Practice

Topical exosome products could be used both short and long terms for skin rejuvenation, scar improvement, hyperpigmentation, and other dermatologic or aesthetic processes. They would provide an alternative for some other popular autologous treatments, such as platelet-rich-plasma (PRP) injections, which are also thought to improve skin appearance through the introduction of growth factors into the interior of the cell. Current studies, such as those included in this systematic literature review, focused on skin outcomes over a period of weeks.^16-25^ There are currently no studies assessing the long-term implications of topical exosome products.

There are currently no studies comparing the effectiveness of topical exosome application to other treatments, such as PRP or laser. Future studies should investigate this in order to establish where this treatment would fall in the lineup of treatments available for skin rejuvenation, in both efficacy and invasiveness.

There are many possible clinical implications of an FDA-approved topical exosome product. These products could be an additional service that plastic surgeons and other providers focusing on aesthetics could add to their arsenal of treatments. These products could be used as a less invasive option for people who are not interested in undergoing procedures, lasers, or injections. Additionally, these products could be offered as a treatment to take in between more invasive treatments, as a maintenance treatment. Finally, exosome availability as a commercial product would be a significant paradigm shift toward the use of autologous products in cosmetics (Table 1).

**Table 1.** Studies Investigating the Use of Topical Exosomes With or Without Combination Treatments

Study	Exosome source	Combination treatments
Ahmadpour et al^13^	Fetal skin fibroblasts	Phosphate-buffered saline (PBS)
Bakhtyar et al^14^	Acellular Wharton's Jelly	PBS
Lu et al^12^	si-ADMSC-EXOs (cell line engineered to secrete exosomes with high levels of siRNA)	PBS
Proffer et al^15^	Human platelet extract	Intensive repair serum (Rion Aesthetics, Rochester, MN)
Ye et al^16^	Mesenchymal stem cells	PBS
Zhang et al^9^	Mesenchymal stem cells	PBS and *Haliclona* spicules
Zhao et al^10^	Human umbilical vein endothelial cells	Gelatin Methacrylol gel
Zheng et al^11^	Adipose stem cells	Botulinum toxin A, Dulbecco's PBS

### Topical Peptides: Proposed Mechanisms of Action

There are 3 main types of peptides currently used in cosmeceuticals—these include signal peptides, neurotransmitter-affecting peptides, and carrier peptides.^38^ Signal peptides are peptides that have the ability to signal fibroblasts to either increase collagen production or decrease collagenase production to slow collagen breakdown. In several studies, which were conducted prior to 2010 and, therefore, excluded from our review, topical application of signal peptides was shown to decrease fine lines and wrinkles significantly compared to placebo alone, and similarly to the application of retinol.^40-42^

Neurotransmitter-affecting peptides include botulinum neurotoxin Type A and peptides that have been developed to imitate this structure. These peptides inhibit the contraction of facial muscles when injected, preventing the formation of fine lines and wrinkles.

Carrier peptides are peptides that are used to deliver and stabilize trace elements that support wound healing and enzymatic function. The most common element delivered by these peptides is copper, which enhances wound healing and angiogenesis. Several of the enzymes essential for collagen production, such as lysyl oxidase, are dependent on copper.^39^ The most common carrier peptide is glycil-L-histidyl-L-lysine, used to transport copper to Collagen I. It has been studied in both wound healing and skin rejuvenation, with the majority of available evidence supporting wound healing.^43-45^

The theory behind topical application of peptides is that when they are combined with a lipophilic carrier, they will be able to permeate the skin barrier. Once past the skin barrier, these peptides will perform their respective modulations to the dermis, through increasing collagen production, inhibiting collagenase, promoting angiogenesis, and wound healing.

### Topical Peptides: Quality and Safety Considerations

Peptides are considered to be very safe therapeutics. They are easily degraded through physiologic enzymatic pathways, giving them a short half-life and little potential for long-lasting negative effects.^8^ Randomized controlled trials are few and far between. Search conducted on October 2, 2023, on Clinicaltrials.gov for “topical peptide” yielded 2 clinical trials investigating topical application of various peptide formulations for cosmetic purposes.^46,47^

Currently, topical peptide therapeutics tend to be designed for frequent use with shorter effect times. This may be due, in part, to the relatively quick degradation of peptide products in the human body, as well as the fact that topical formulations are exposed to weather, sweat, sun, and soap, decreasing their ability to remain on the surface of the skin for a lengthy period. In order for a topical peptide formulation to have longer lasting effects, it would likely have to be in a slow-release formula that is able to penetrate the skin barrier effectively without immediately diluting in the bloodstream.

### Implications of Topical Peptides in Practice

The introduction of topical peptide products into the plastic surgeon's practice, perhaps as an adjunct to other aesthetic procedures, such as injectables or cosmetic surgery, could help improve patient experience and preserve cosmetic outcomes for much longer. Topical peptide products applied after botox could hypothetically extend the lifespan of the injection and provide further cosmetic benefit by improving skin plumpness and texture. The use of these products in conjunction with available aesthetic interventions could improve patient satisfaction and potentially lead to overall better outcomes.

The advent of topical peptides as a therapy for skin rejuvenation would have the greatest impact on dermatology, aestheticians, and plastic surgery. However, although many products have the capability to reverse the visual signs of skin aging, they often work for only brief intervals at a time and must be applied up to twice daily to maintain effect. Although topical application is easier to access and use, the high burden of use frequency may lead to many people choosing interventions to improve skin rejuvenation that have a longer lasting effect, such as laser therapy or fat grafting (Table 2).

**Table 2.** Studies Investigating the Use of Topical Peptide Therapy With or Without Combination Treatments

Study	Peptides used	Combination treatments
Akulinina et al^17^	Derived from rice and lupin	Liftactiv peptide C ampoules, Laboratoires Vichy, France (pure Vitamin C, hyaluronic acid, mineralizing water)
Fossa Shirata and Maia Campos^18^	Rice	Sunscreen and gel cream formulation containing ascorbyl tetraisopalmitate
Hu et al^19^	Collagen peptides from skin of Nile tilapia	Not described
Jeong et al^20^	Undefined peptides	Not described
Kong et al^21^	Cod skin collagen peptides	Gel created from acetic acid, beta-glycerophosphate powder
Maia Campos et al^22^	Hydrolyzed di- and tri-peptide proteins of rice	Hydroxyethylcellulose, methylphenyl polysiloxane, cyclomethicone, cyclomethicone and crosspolymer dimethicone, hydrosoluble filter UVA/UVB, propylene glycol, glycerin, 2-phenoxyethanol, methylisothiazolinone, water
Mo et al^23^	Extracted from rice fermentation of *Lactobacillus plantarum*	Water
Park et al^24^	Novel peptide derivative comprising laminin 5	Baum L.C.E. (France, Laboratories D’Anjou)
Peng et al^25^	Oyster peptides	Deionized water
Qin et al^26^	Prohealing peptide RL-QN15	Zinc alginate hydrogel

### Landscape of FDA Approval for Topical Exosome and Topical Peptide Products

The pathway to FDA approval has many steps. Generally, new therapeutics begin with preclinical testing, to assess safety, then move on to investigational new drug applications. Once these are approved, clinical trials may begin. Once clinical trials confirm safety and effectiveness, the manufacturer may submit a new drug application; after the new drug application has been received, all of the available data are reviewed by the FDA and they pass a verdict on whether the drug will be FDA approved or not. The average length of time from submission of a new drug application to FDA approval is about 6 to 10 months.

There are currently no FDA-approved topical peptide or exosome products commercially available. One of the difficulties of investigating skin rejuvenation products is the multitude and variability of different primary outcomes to be investigated, such as overall skin appearance, skin elasticity, photoaging, etc. Additionally, many of these endpoints are subjective and dependent on the rating of the study team. Ideally, future clinical trials investigating the efficacy of topical peptides and topical exosomes for skin rejuvenation would identify at least 1 primary objective endpoint to assess outcomes.

There are many current clinical trials investigating the effectiveness of topical exosomes in treating a myriad of dermatologic conditions as well as the effects of exosomes on skin rejuvenation. In addition to proving efficacy of topical exosomes for skin rejuvenation and elucidating possible adverse effects, exosome manufacturers will likely have to address the unique concerns for exosome production, including quality control, dosing, and production concerns. Additionally, because exosomes may be derived from stem cells, extra considerations and approval may be required.

Many topical peptide formulations currently on the market contain small di- or tri-peptides suspended in a moisturizing solution and packaged with other therapeutic ingredients, such as vitamin C, hyaluronic acid, or retinoids.^17,18,22^ As topical peptides have been used in cosmeceuticals for some time, that are not FDA regulated, manufacturers will likely be able to begin with randomized controlled clinical trials in order to establish effectiveness before applying for FDA approval. There will likely not be a need to establish safety as these products are already proven safe for consumers. This provides the groundwork for a relatively quick and straightforward path to FDA approval, as long as the investigational products are proven to be effective in a randomized, controlled trial setting.

The paucity of evidence demonstrating the effectiveness of topical peptides in a controlled setting is a potential barrier to FDA approval. This gap in research may be due to the ability of cosmeceuticals to bypass FDA approval, reducing the incentive for pharmaceutical companies to fund clinical trials investigating these products.

FDA approval of topical products for skin rejuvenation would lend another tool for plastic surgeons, dermatologists, and aestheticians to offer to patients who desire skin rejuvenation but may be opposed to or have clinical contraindications to current therapies, such as injectables or laser therapy. Additionally, as no FDA-approved topicals are currently on the market, the first drug to achieve this designation would be uncontested in the marketplace.

### Limitations of this Review

The major limitation of this systematic review is that it investigates experimental products that are not currently FDA approved, and therefore, there are few available articles and trials. If FDA approval for either topical exosomes or peptides was to be achieved, this would open the door for future studies further investigating effectiveness.

## CONCLUSIONS

The future of topical exosome and topical peptide products for the purpose of skin rejuvenation appears promising. There are a plethora of clinical trials currently in progress to evaluate the safety and effectiveness of exosomes for various dermatologic treatments, in addition to the significant research exploring exosomes' role in wound healing. The biggest challenge that these products must overcome in order to gain FDA approval is standardizing exosome production and addressing in vivo stability, as well as continuing to deepen our understanding of the mechanism of action of exosomes at a biochemical level. Once this is achieved, we may see topical exosome products on the market in the near future.

It is difficult to be specific about the future potential of topical peptide therapeutics for skin rejuvenation because the array of peptides available or able to be formulated in a lab is quite broad. The topical peptide formulations that have been formally investigated are not dangerous to human health and have few to no negative side effects. There have been few trials conducted to assess the efficacy of topical peptides; it seems that many trials have focused mainly on safety. Although there is not yet an FDA-approved formulation for topical exosomes or topical peptides, peptides are plentiful in so-called “cosmeceuticals” and may be purchased at any drugstore or online. For a topical peptide product to truly pave the way for FDA approval and regulation, the authors postulate that it would have to have quite a significant impact that is not currently available from commercial cosmeceutical topical peptide preparations.
